# Persistency of the Urachus1Read before the Illinois Veterinary Medical and Surgical Association, Jan. 14 and 15, 1903.

**Published:** 1903-02

**Authors:** S. H. Swain

**Affiliations:** Decatur, Ill.


### PERSISTENCY OF THE URACHUS.[1]

By S. H. Swain, V.S.,
DECATUR, ILL.

The urachus is a narrow canal or duct extending from the urinary bladder through the umbilicus to the allantoid sac, where the urinary secretions are discharged and retained until the termination of gestation.

The indications of persistency of the urachus are shown by an escape of urine at the umbilicus shortly after birth. At the termination of gestation nature has intended that this canal of escape for the urine through the urachus should be completely occluded. The escape of urine should then be changed to the urethra; but it

[1] Read before the Illinois Veterinary Medical and Surgical Association, Jan. 14 and 15, 1903.

sometimes happens that nature has not quite completed her work, and the urine continues after birth to escape through the urachus or through both the urachus and urethral canal, in which case, if immediate and proper attention is not given the case, the escape of urine through the urachus at the navel will result in omphalitis, and is usually followed by septic infection, arthritis, and death.

The equine family seems much more subject to this abnormality than the bovine or other domestic animals, and the draught breeds of horses more subject than the finer breeds; the male foal more subject to this persistency than the female.

*Treatment.* The surgeon should first ascertain whether or not the urethral canal is pervious. This may be definitely ascertained by patiently watching the young creature for a short time until an effort is made to urinate, when, if it is seen that no urine is passed by the urethral outlet, the surgeon should at once endeavor to pass a suitable catheter. This being satisfactorily accomplished, or if the above observation should satisfy the surgeon that the urine is passed through the normal outlet as well as through the urachus, then he should proceed to treat the abnormality, first by cleansing the umbilicus and also the internal surface of the urachus. This may be accomplished by injections carefully made into the urachus by means of a suitable syringe of antiseptic and astringent solution until the surgeon is satisfied that the parts are thoroughly cleansed. Probably one of the best preparations for this purpose is composed of a 1 per cent. solution of formalin, with thirty grains of powdered alum added to each ounce of the solution. This should be repeatedly injected into the urachus until its cavity is thoroughly cleansed. Then the surgeon should proceed to close the urachus, and this may be accomplished by ligature if the urachus has been severed an inch or more external to the navel; but if separation of the urachus has taken place very near or within the umbilicus I would much prefer local astringent and antiseptic remedies to either ligatures or sutures; and for this local treatment I would advise the following, which has proven very satisfactory in my practice: iodoform, one ounce; powdered alum, one ounce; boric acid, four ounces; mixed and applied morning, noon, and night until the urachus is closed and the umbilical wound healed. This antiseptic treatment should be applied, even if closure of the urachus has been effected by ligature; and if the patient is exhibiting symptoms of septic infection an effort should at once be made to combat this trouble, and would advise the internal administration of the following: acid carbolic, one drachm; glycerin, seven drachms; water, eleven ounces. Mix and give one ounce in eight ounces of water every four hours during the day as long as required; and as a local application to the inflamed and swollen joints, two ounces of compound solution of iodine and iodide of potassium to fourteen ounces of water. This should be freely applied three times daily as long as is necessary.

